# Causes of Deceased Donors Loss before Organ Retrieval

**Published:** 2018-03

**Authors:** Meysam Mojtabaee, Shahrzad Ghaffarian, Shagin Shahryari, Farahnaz Sadegh Beigee

**Affiliations:** Lung Transplantation Research Center (LTRC), National Research Institute of Tuberculosis and Lung Diseases (NRITLD), Shahid Beheshti University of Medical Sciences, Tehran, Iran.

**Keywords:** Organ donation, Brain death, Diabetes insipidus, Deceased donor

## Abstract

**Background:**

When potential brain dead donors are in line-up for organ retrieval, their loss would be such a disaster. The aim of this study was to detect the occurrence of different disorders leading to pre-retrieval donor's cardiac arrest and loss in order to prevent this energy and money wasting challenge.

**Materials and Methods:**

In this observational study, medical records of potential donors from 2001 to 2016 who were lost after transfer to Organ Procurement Unit (OPU) of Masih Daneshvari Hospital and before organ donation were reviewed and weigh of every responsible disorder was tested. Equal number of actual organ donors were randomly selected others for comparison.

**Results:**

In 14 years of experience in organ donation, 46 (3.09%) out of 1485 potential donors were lost after their transfer to OPU with the aim of organ donation. Mean age of donors and their gender were not significantly different to actual donors (37.4 ± 17.7 versus 39.2 ± 18.4, P= 0.2). However, proportion of drug toxicity as the cause of brain death was more common in the lost donors (19.5 versus 5.3%, P= 0.001). Thirteen (28.2%) of the cases had a documented history of ischemic heart disease, which was not as common in actual donors. After excluding hypotension and diabetes insipidus, more incident disorders among the lost donors were metabolic acidosis, hypocalcaemia, hyperglycemia, thrombocytopenia, severe anemia and different presentations of coagulopathy. Clinical conditions of 47.8% of cases were flared up by different severities of acute kidney injury and mean ALT levels were significantly higher than actual donors. All the above mentioned disorders were significantly more common in lost donors than actual ones.

**Conclusion:**

Drug toxicity, history of ischemic heart disease and occurrence of acute kidney injury are associated with more potential donors' loss before organ retrieval.

## INTRODUCTION

Transplantation is still the last rescue treatment for many solid organ end-stage diseases (1). Currently, the best accessible and acceptable sources of these organs are deceased donors. However, unprecedented scarcity of transplantable organs have solicited enhanced effort to expand donor pool (2). Critical management of brain-dead donors have been proven to increase chances of conversion of possible donors to actual ones (3), increase number of organs donated per donor (3) and also improve post-transplant outcome (4).

Pathophysiologic process of brain death has been reported frequently to cause the donors' hemodynamic instability and organ damage. Additionally, Wilhelm MJ et al. discovered that 10–20% of patients undergoing catecholamine storm in the course of brain death would be facing cardiac arrest before full-blown loss of brain function (5). Therefore, circulatory survivors will get severe harmful assaults from the process which can cause metabolic, hematologic and coagulation disorders as well as fluid and electrolyte disturbances (6) before organ retrieval. Loss of pituitary function can lead to Antidiuretic Hormone (ADH) insufficiency and Diabetes Insipidus (DI). The latter situation would cause polyuria, hypernatremia and other electrolyte imbalances besides arterial hypotension due to loss of intravascular volume (7). Lack of thermoregulation and the consequent hypothermia would be accompanied by metabolic acidosis and coagulopathy (8). Being prone to infectious agents, the potential donors may develop sepsis (9). Hormonal insufficiency might lead to inappropriate metabolism of organs and circulatory collapse (9). The above mentioned conditions as well as many other ICU-associated complications can result in making the efforts of organ donation process in vain.

When potential brain dead donors are in line-up for organ retrieval, their loss would be such a disaster to come. The aim of this study was to detect and evaluate the occurrence of different disorders leading to pre-retrieval donor's cardiac arrest and loss in order to prevent this energy and money wasting challenge.

## MATERIALS AND METHODS

In the 14 years of organ donation practice from 2003 to 2016, when primary diagnosis was made, potential donors got transferred by an ambulance from the original care-giver hospital to ICU of Organ Procurement Unit (OPU) of Masih Daneshvari Hospital. While completion of formal process of brain death certification and obtaining donation consent from next of kin, medical management of the donor continued to dispel laboratory abnormalities as well as clinical casualties. Demographic data (age and sex), history of any disease such as ischemic heart disease or organ failure, cause of brain death, type of misused agent in case of drug toxicity, any drugs administered in treatment process, hospitalization and intubation period, and need for inotropic agents were among the provided record for every donor. Prevalence of hypo/hypertension, electrolyte disturbances (Sodium, Calcium, Potassium, Magnesium, and Phosphate), DI metabolic disorders (acidosis or alkalosis), hematologic disorders (anemia, thrombocytopenia, and leukopenia/cytosis), coagulopathy or renal/liver failure were recorded.

In this retrospective study, the above factors and abnormalities were compared between the potential donors who were lost before organ retrieval surgery to 46 actual donors (equal quantity). The compared groups were selected randomly from list of donors regarding their age. With every lost potential donor, an actual one was selected with ±2 years as the accepted difference. Among the equivalent cases, one of them was randomly selected. Statistical analyses were performed, by SPSS software version 23. The quantitative data was reported as mean ± standard deviation (SD). The nominal variables were presented as percentages. Subsequently, student's sample t-test or Chi-square with significance set at (P ≤ 0.05) was used to analyze the data.

## RESULTS

In 14 years of experience in organ donation from 2003 to 2016, 46 (3.09%) out of 1485 potential donors were lost after their transfer to OPU with the aim of organ donation. Mean age of donors were not significantly different to actual donors (37.4 ± 17.7 versus 39.2 ± 18.4 years, respectively). Similarly gender proportions were not significantly different in the two groups (Male=59 vs. 57.5%, respectively) However, proportion of drug poisoning as the cause of brain death was more common in the lost donors (19.5 versus 5.3%, P=0.001). Mean age of poisoned cases (actual donors and lost ones) was 26.7 ±14.6 years. On the other hand, mean age of trauma-suffered cases was 29.4 ± 17.3 years. In contrast, Cerebrovascular Accident (CVA) associated brain death victims had a mean age of 54.1 ± 8.6 years.

13(28.2%) cases had a documented history of ischemic heart disease, which was not as common in actual donors (4 cases, 8.6%, P=0.02). Table 1 summarized the donors' demographics factors.

**Table 1.** All candidate donors' demographics and related factors.

	**Actual donors**	**Lost donors**	**P-Value**
**Age**	39.2 ± 18.4	37.4 ± 17.7	N.S
**Sex (male)**	57.5%	59%	N.S
**History of IHD**	8.6%	21.7%	0.02
**Cause of Brain Death (trauma)**	57%	60%	N.S
**Cause of Brain Death (toxicity)**	5.3%	19.5%	<0.001
**Intubation (days)**	5.8 ± 6.2	6.5 ± 5.7	N.S
**Smoking history (P.Y)**	8 ± 10	6.5 ± 10	N.S
**Mean Arterial Blood Pressure**	82.2 mmHg	58.9 mmHg	<0.01
**Central Venous Pressure**	12.7 ± 3.4	14.8 ± 3.1	N.S

N.S: Not significant

After excluding hypotension and DI which are two common stations in natural course of brain death (10), the leading disorders among lost cases were metabolic acidosis in 30 cases (65.2%), hypocalcaemia in 22 cases (47.8%), hyperglycemia in 21 cases (45.6%), thrombocytopenia in 36 cases (78.2%), different presentations of coagulopathy in 18 cases (39.1%) and severe anemia in 19 cases (41.3%).

8 out 9 (88.85%) poisoned lost cases had metabolic acidosis, which was higher than the other ones (59.4%, P<0.01). Clinical conditions of 22 (47.8%) cases were flared up by different severities of acute kidney injury and mean ALT levels were significantly higher than actual donors (286.2 versus 105.6, P<0.001). All the above mentioned disorders were significantly more common in lost donors than actual ones. Table 2 describes comparison of the disorders between the two groups of cases.

**Table 2.** Incidence of laboratory abnormalities among two groups of cases

**Laboratory abnormality**	**Actual donors**	**Lost potential donors**	**P-Value**
**Metabolic acidosis**	17.3%	65.2%	<0.001
**Hypocalcaemia**	30.4%	47.8%	0.05
**Hyperglycemia**	23.9%	45.6%	0.03
**Coagulopathy**	15.2%	39.1%	0.01
**AKI**	6.5%	47.8%	<0.001
**Mean ALT**	105.6	286.2	<0.001
^*^Thrombocytopenia	39.1%	78.2%	0.02
^**^Severe anemia	13%	41.3%	<0.001
**Hyperkalemia**	15.2%	19.5%	N.S
**Metabolic alkalosis**	8.6%	4.3%	N.S
**Respiratory acidosis**	15.2%	13%	N.S
**Hypernatremia**	91.3%	93.4%	N.S
**Hypokalemia**	21.7%	24%	N.S
**Hypophosphatemia**	45.6%	41.3%	N.S
**Hypomagnesaemia**	17.3%	15.2%	N.S

N.S: Not significant

* Thrombocytopenia: Plt< 100,000/microliter,

** Severe anemia: Hb< 7 mg/dl

## DISCUSSION

Results of the study suggest that incidence of metabolic acidosis, hypocalcaemia, hyperglycemia, coagulopathy and organ failure (kidneys, liver and lungs) are higher in lost brain-dead potential donors. After being admitted to the specialized ICU, critical management of the donor continues to warrantee optimal management goals. The literature is rich in published data reporting the substantial benefits of proper donor management (11) and avoidance of rushing into organ retrieval (12) in outcome of transplanted organs.

Jansen et al. reported that after family refusal (57–62%), donor management problems and surgical techniques (38–43%) were crucial setbacks in way of conversion of potential donors into actual ones in Netherlands (13). We were not able to find any alike study in our country or in any similar organ donation organizations with the same strategy regarding proportion of donor loss after their transfer.

Increasing donors' age may be a relatively new difficulty for our OPU. We have had a history of organ procurement from young trauma-suffered donors in the past. Najafizadeh et al. reported mean age of 31.7 ± 15.5 years in 2009 (14). Kazemeyni and Aghighi introduced the mean age of 29 years in a population of their donors (15). Nowadays, prominent proportion of donors with CVA as the cause of brain death is getting close to statistics of developed countries (16). Elderly donors which are starting to be the leading group of deceased ones (17), are bringing heavier loads of cardiovascular risk factors as well as history of ischemic heart disease to the circle of donors. However, existence of toxicity in a relatively high proportion of lost candidates keeps the overall mean age of theirs equal to actual donors, as we can see that mean age of the lost poisoned cases are 26.7 years. Acute poisoning of different agents may be responsible for more incidences of certain conditions such as metabolic acidosis. These explanations leave elderly and poisoned potential donors more prone to loss before organ donation surgery.

We witnessed both hypotension and DI a pandemic condition in our cases. However, Fiser et al. reported DI in only 38% of children with brain death in autopsies (18). Gramm et al. found 78% of cases to be suffering from DI (19). As mentioned before, these two disorders have been proven to occur in all experimental brain death models. One possible explanation may be the fact that some other human based studies considering lower occurrence of the conditions have included all brain-dead cases who may or may not become organ donors. As opposed to, we observed final candidates for organ donation. Therefore we were facing full-blown progress of brain death because we took time for donor management.

Acute Kidney Injury (AKI), liver function tests abnormalities and acute respiratory distress syndrome which is defined by low PaO_2_/FIO_2_ as well as clinical criteria, were observed more in lost donors. The findings regarding high mortality rate of ICU hospitalized patients with organ failure is not new (20–22). In our insight, donors with single transplantable organs are more prone to irreversible cardiac arrest and loss. The fact defines need for more prompt management protocols for these potential donors.

## CONCLUSION

Drug toxicity as the cause of brain death, history of ischemic heart disease and occurrence of acute kidney injury and liver and lung dysfunction are associated with more potential donors' loss before organ retrieval. Metabolic acidosis, hyperglycemia, hypocalcaemia and coagulopathy are more common in unsuccessful organ donation effort. As a result, implementation of proper management guidelines, especially regarding these abnormalities is recommended.
